# Culturally-consistent diet among individuals of Mexican descent at the US-Mexico border is associated with sleep duration and snoring

**DOI:** 10.1186/s40795-021-00452-0

**Published:** 2021-08-23

**Authors:** Sadia B. Ghani, Krishna Taneja, Chloe C. A. Wills, Andrew S. Tubbs, Marcos E. Delgadillo, Dora Valencia, Mohamed Halane, William D. S. Killgore, Michael A. Grandner

**Affiliations:** 1 Department of Psychiatry, Sleep and Health Research Program, University of Arizona, 1501 N Campbell Rd Suite 7326, Tucson, AZ 85724-5002 USA; 2 Medical University of the Americas, Nevis, Saint Kitts And Nevis; 3 Department of Psychiatry, Social, Cognitive, and Affective Neuroscience (SCAN) Lab, University of Arizona, 1501 N Campbell Rd Suite 7303B, Tucson, AZ 85724 USA

**Keywords:** Sleep duration, Cultural food, Sleep health, Snoring, Border health disparities

## Abstract

**Background:**

Existing studies show that consuming food consistent with one’s culture reduces cardiometabolic risk. However, few studies have assessed whether these dietary choices influence sleep health. Accordingly, this study assessed how Mexican food consumption by individuals of Mexican descent residing at the US-Mexico border, was associated with various measures of sleep, after accounting for acculturation.

**Methods:**

Data were provided by 100 adults between the ages of 18–60, in the city of Nogales, AZ. Questionnaires were provided in either Spanish or English. Acculturation was assessed with the Acculturation Scale for Mexican-Americans (ARSMA-II), with an additional question, asking how often “my family cooks Mexican foods.” Frequency of cooking Mexican food was coded as either “yes” or “no.” Sleep was assessed, using validated measures that include the Insomnia Severity Index (ISI), the Epworth Sleepiness Scale (ESS), the Pittsburgh Sleep Quality Index (PSQI), and sleep duration with the item “how many hours of actual sleep did you get at night?” Regression models estimated the associations between sleep health variables as outcomes and consumption of Mexican food as the independent variable. Covariates included age, sex, and acculturation scores. Parental education level was also included, as an indicator of childhood socioeconomic status and since food culture likely involves parents.

**Result:**

We found that among individuals who identified as Mexican-Americans who consumed culturally-consistent foods, was associated with, on average, 1.41 more hours of sleep (95% CI 0.19, 2.62; *p* = 0.024) and were less likely to report snoring (OR: 0.25; 95% CI 0.07, 0.93; *p* = 0.039). Consuming Mexican food was not associated with sleep quality, insomnia severity or sleepiness.

**Conclusion:**

Individuals of Mexican descent residing at the US-Mexico border who regularly consumed Mexican food, reported more sleep and less snoring. Mexican acculturation has been shown previously to improve sleep health. This is likely due to consumption of a culturally- consistent diet. Future studies should examine the role of acculturation in sleep health, dietary choices, and subsequent cardiometabolic risk.

## Introduction

Previous studies have shown that long and short sleep duration is related to dietary nutrient and timing of intake [1–5]. Laboratory studies show that alterations in sleep duration and timing of sleep can result in acute changes to dietary intake patterns, including increased daily caloric intake and meal consumption and reduced energy expenditure [6, 7]. Additional work at the community and population level shows that insufficient sleep duration and/or poor sleep quality are associated with unhealthy food intake, including preferring fatty foods, skipping breakfast, snacking, eating outside the home [1, 3, 8, 9], weight gain [10], and incident obesity [11, 12], even after adjusting for BMI and physical activity [3]. While informative, prior studies have not specifically addressed how sleep may influence dietary intake in racial/ethnic minorities, particularly Hispanic/Latino individuals.

Obesity is a rising public health epidemic in the United States (US) [13, 14], and rates of obesity are disproportionately higher in Hispanic individuals [15, 16], particularly Mexican-Americans [13]. Hispanics/Latinos are now the largest minority group in the US and Mexican-Americans are the largest sub-group within this population [17], so addressing this disparity should include a focus on sleep health, as it relates to food intake. Sleep disparities have been documented in Hispanics/Latinos as well [18, 19], and emerging evidence suggests that habitual sleep is decreasing among Mexican-Americans at more than twice the rate seen in Non-Hispanic Whites [20]. A better understanding of the relationships between sleep and food intake in Mexican-Americans may help address these disparities.

Mexican-Americans exhibit more unhealthy cardiometabolic outcomes when they adopt an “Americanized” dietary pattern, including weight gain [21], obesity [22, 23], and risk of diabetes [23]. These outcomes are also associated with poor sleep in this population [13, 16, 18, 24–29], such that adopting an “Americanized” diet may be related to poor sleep. Alternatively, poor sleep may influence poor dietary choices [1, 2, 6, 30] and acculturation may contribute to this relationship [31–33]. Acculturation towards an “Americanized” mindset is associated with a greater likelihood of insufficient and/or poor-quality sleep [31–35], while less acculturation may protect against poor sleep and negative health behaviors [31, 32]. Although eating more culturally-consistent food is associated with better health outcomes in this population [36], the specific relationships between sleep and culturally-consistent diets have not been explored, especially in the Hispanic/Latino population. If eating culturally-consistent foods is associated with better sleep quality independent of overall acculturation, then interventions targeting sleep and culturally-consistent diets may reduce cardiometabolic disease in the Mexican-American population.

Accordingly, the present study evaluated whether habitual consumption of Mexican food by individuals of Mexican descent living at the US-Mexico border was associated with sleep duration, sleep quality, daytime sleepiness and snoring, independent of acculturation. The hypotheses were that individuals from this group who do not consume these foods would report (1) shorter habitual sleep duration, (2) worse sleep quality, (3) more daytime sleepiness, and (4) more snoring.

## Methods

### Participants

A sample of *N* = 100 adults between the ages 18–60, of Mexican descent living in Nogales, AZ were surveyed for this study. Recruitment involved in-person solicitations, flyers, and social media posts in both English and Spanish. Participants were (1) fluent in English or Spanish, (2) over the age of 18 years, and (3) identified as Mexican-American or Mexican. Exclusion criteria included medical conditions or serious mental illnesses that affected the participant’s ability to provide consent or participate. Surveys were distributed in the participants preferred language, via a tablet and administered by a fully bilingual research staff during late summer and early fall of 2017. The study protocol was approved by the University of Arizona IRB (protocol # 1608763580) and informed consent was obtained prior to participation. All methods were carried out in accordance to relevant regulations and guidelines.

### Measures

The Pittsburgh Sleep Quality Index (PSQI) [37] assessed overall sleep quality. The PSQI is well validated and frequently used screening tool, assessing several domains of sleep and differentiates “good” and “poor” sleepers. A summed total score greater than 5 is indicative of worse sleep quality [37]. The PSQI has been previously used in this population [31].

The Insomnia Severity Index (ISI) [38] assessed both daytime and nighttime components of insomnia. It is a well-recognized and validated index and is used as a standard outcome measure in studies related to insomnia [38]. It is available in several languages including Spanish [39], and has been previously used in this population [31].

Sleep duration was assessed by asking participants: “How many hours of actual sleep did you get at night?” (This may be different than the number of hours you spend in bed).” Responses were recorded in 15-min increments.

Daytime sleepiness was assessed using the Epworth Sleepiness Scale (ESS) [40], which is validated in clinical and research settings [41], is available in Spanish [42], and has been previously used in this population [31]. The ESS measures the propensity to fall asleep in a range of situations with higher scores indicating increased daytime sleepiness [40].

Participants were asked to state how often they snored with options including “never,” “rarely,” “sometimes,” “often,” or “always.”

Acculturation was assessed with the Acculturation Rating Scale for Mexican Americans II (ARSMA-II) [43]. This scale includes two independent subscales for “Mexican” and “Anglo” acculturation, and it includes 13 questions on the Anglo Orientation Subscale and 17 questions on the Mexican Orientation Subscale. Respondents answer each question on a Likert scale ranging from 0 (“not at all”) to 4 (“extremely often or almost always”), and responses are averaged within each subscale. The higher the value on each scale indicates the higher acculturation of either Mexican and/or Anglo acculturation.

The degree to which individuals report eating culturally-consistent food was assessed with the survey item, “My family cooks Mexican Foods.” Responses were coded as “yes” if individuals answered “very often” or “almost always” and “no” if individuals answered “not at all,” “not very often,” or “moderately.”

Finally, age, sex, acculturation and parental education levels were included as covariates since parental education is an indicator of childhood socioeconomic status and because food culture likely involves parental influence.

### Translation of measures into Spanish

Wherever possible, previously validated Spanish versions of questionnaires were used. English-only measures were translated using Guideline-based translation procedures [44, 45]. Specific translation procedures are described in detail elsewhere [31], including some textual changes to the ISI to accommodate regional language preferences [31]. Ultimately, all questionnaires were available in either English or Spanish and participants completed these measures in the language of their choice.

### Statistical analyses

The study outcomes were sleep duration (hours), sleep quality (PSQI score), insomnia severity (ISI score), daytime sleepiness (ESS score), and snoring. All variables were continuous except for snoring which was treated as an ordinal variable. The independent variable was consumption of Mexican food (yes/no), and sex, age, parental education, and acculturation were included as covariates. Linear and ordinal logistic regression models evaluated the relationships between consumption of Mexican food and each sleep variable. A likelihood ratio test comparing ordinal and binomial logistic regression models found no significant differences (*p =* 0.3), suggesting that the ordinal approach was appropriate. Results are reported as unstandardized regression coefficients (B) for linear variables and ordinal Odds Ratios (OR) for snoring, with 95% confidence intervals (CI). All analyses were performed in STATA 14.2 (STATACORP, College Station, TX).

## Results

Table 1 reports the characteristics of the sample. Most respondents (*N* = 87) reported that their family regularly prepared Mexican food. The mean age was 36.5 years (SD = 19.1), and the sample was 47% female. Mean Mexican acculturation was 2.90 (SD = 0.75) and mean Anglo acculturation was 1.94 (SD = 0.94), indicating participants had stronger orientation toward Mexican acculturation. A plurality of respondents reported a parental education less than a high school. Mean sleep duration was 6.06 h (SD = 1.75) and the mean PSQI score was 7.73 (SD = 3.47) indicating poor sleep quality. The mean ISI score was 9.14 (SD = 4.20), which falls in the range of subclinical insomnia, and the mean ESS score was 6.36 (SD = 4.33), which indicates typical levels of sleepiness. Finally, 41% of respondents reported “never” snoring while, 13% reported “always” snoring. Those who consistently ate Mexican food reported higher levels of Mexican acculturation (*p* = 0.0004) and longer sleep durations (*p* = 0.0377) than those who did not. There were no other significant differences between groups.

**Table 1:** Characteristics of the *N* = 100 Individuals; Late Summer/Early Fall 2017; Nogales AZ

^**a**^Variable	Complete Sample (***N =*** 100)	Stratified by Frequent Consumption of Culturally-Consistent Food		
		No (***N*** = 13)	Yes (***N*** = 87)	^**a,b**^***p***
Age (Years)	36.50 ± 19.10	30.34 ± 23.07	37.45 ± 18.36	0.3050
Sex				0.9480
Male	53.00	53.85	52.87	
Female	47.00	46.15	47.13	
Acculturation				
Mexican	2.90 ± 0.75	1.79 ± 0.96	3.06 ± 0.55	0.0004
Anglo	1.94 ± 0.94	2.03 ± 1.00	1.93 ± 0.93	0.7449
Mother’s Education				0.7924
Less Than High School	42.00	30.80	43.70	
High School	13.00	15.40	12.60	
Some College	23.0	23.10	23.00	
College	22.0	30.80	20.70	
Father’s Education				0.3120
Less than High School	39.00	23.10	41.40	
High School	18.00	30.80	16.10	
Some College	20.00	30.80	18.40	
College	23.00	15.40	24.10	
PSQI Sleep Duration (Hours)	6.06 ± 1.75	4.83 ± 2.14	6.24 ± 1.62	0.0377
PSQI Total Score	7.73 ± 3.47	7.69 ± 4.42	7.74 ± 3.34	0.9734
ISI Score	9.14 ± 4.20	8.08 ± 4.52	9.30 ± 4.16	0.3726
ESS Score	6.36 ± 4.33	7.00 ± 5.58	6.26 ± 4.14	0.6549
Snoring				0.0681
Never	41.00	30.80	42.50	
Rarely	18.00	15.40	18.40	
Sometimes	19.00	15.40	19.50	
Often	9.00	30.80	5.75	
Always	13.00	7.69	13.80	

^a^Values reported as percent or mean ± standard deviation (% or M ± SD)

^b^Tests of significance were either student’s t-tests or chi-squared tests

*PSQI* Pittsburgh Sleep Quality Index, *ISI* Insomnia Severity Index, *ESS* Epworth Sleepiness Scale

Table 2 reports the associations between consumption of Mexican food and sleep related variables. In adjusted analyses, those whose family regularly made Mexican food reported 1.41 h of sleep (95% CI: 0.19, 2.62, *p* = 0.024), and were less likely to report snoring (OR: 0.25, 95% CI: 0.07, 0.93, *p* = 0.039). No statistically significant differences were seen for sleep quality, insomnia severity, or daytime sleepiness.

**Table 2:** Associations between Frequent Consumption of Mexican Food and Sleep-Related Variables Among Individuals of Mexican Descent; *N =* 100; Late Summer/Early Fall 2017; Nogales AZ

*N* = 100	Sleep Duration (hours)			Sleep Quality (PSQI score)			Insomnia Severity (ISI score)			Sleepiness (ESS score)			Snoring		
Mexican Diet	B	95% CI	***p***	B	95% CI	***p***	B	95% CI	***p***	B	95% CI	***p***	OR	95% CI	***p***
No	Reference			Reference			Reference			Reference			Reference		
Yes	1.41	0.19, 2.62	0.0240	0.11	−2.27, 2.48	0.9290	1.93	−0.95, 4.81	0.1860	−0.89	−4.08, 2.31	0.5810	0.25	0.07, 0.93	0.0390

Models adjusted for age, sex, maternal education, paternal education, Mexican acculturation, and Anglo acculturation

*OR* Odds Ratio, *CI* Confidence Interval, *PSQI* Pittsburgh Sleep Quality Index, *ISI* Insomnia Severity Index, *ESS* Epworth Sleepiness Scale

*P* < 0.05 considered statistically significant

## Discussion

The present study explored how a culturally-consistent diet related to sleep duration, insomnia severity, daytime sleepiness, sleep quality, and snoring in adults of Mexican descent at the US-Mexico border. Overall, a culturally-consistent diet was associated with longer sleep duration and decreased likelihood of snoring, but was not associated with daytime sleepiness, insomnia, or sleep quality.

The main finding was that adults of Mexican descent who primarily ate Mexican food reported 1.41 more hours of sleep compared to those who did not eat Mexican food. Social and cultural factors contribute to the determinants of sleep health [46, 47], and food choice represents one cultural element which has clear health implications [48, 49]. These findings support previous work showing that culturally traditional diets (e.g. Mediterranean, Lebanese, Nordic, and Okinawan diet) lead to better health outcomes [50–54], possibly because of the large amounts of plant-based foods, vegetables, and fruits, and the low amounts of animal-based foods (e.g. red meat), fewer additives, and less refined sugars [50–54]. Studies show that these diets reduce the risk of hypertension and cardiovascular disease, while the western diet is associated with cardiometabolic risk [55, 56]. Additionally, consumption of traditional Mexican food reduces the risk of pre-diabetes [57] and lowers insulin levels [58], although effects on incident obesity are mixed [59–61]. While few studies have explored sleep and the Mexican diet, sleep is closely related to diet and metabolism generally [62, 63] and so the Mexican diet likely has an effect on sleep. Further exploration is necessary to understand the role of culturally-consistent diets on sleep health due to the paucity of available literature. Additionally, the novel findings reported here may guide future sleep health interventions to reduce cardiometabolic disorders and improve health disparities in this population. Future studies should evaluate whether a traditional Mexican diet may improve sleep and other health outcomes in this and other populations.

Acculturation strongly influences food choices because nutrition-related attitudes and behaviors tend to be established early in life and are primarily determined by a combination of cultural, psychosocial and socioeconomic factors [64, 65]. Many aspects of food preparation, purchasing, and eating habits are culturally defined, and individuals may either consciously or unconsciously participate in these activities to preserve traditions and maintain group identity. Acculturation associated with sleep health [31–34] and lower Anglo acculturation may protect against poor sleep quality [18, 19, 34, 35]. Furthermore, greater degree of Anglo acculturation is associated with unhealthy weight gain [22], lower rates of physical activity, higher rates of fast-food consumption [66], and increased rates of alcohol use and smoking [67]. Adapting to western culture may lead to Americanized dietary choices, in turn leading to negative cardiometabolic health outcomes [21–23] and greater likelihood of insufficient sleep and poor sleep quality [31–35]. In this study, although those with higher Mexican acculturation scores were more likely to eat Mexican food, dietary choices are just one part of acculturation. The correlation between Mexican acculturation and eating culturally-consistent food was 0.47, which is high but does not meet the threshold of meaningful collinearity. Therefore, acculturation was included as a covariate.

Parental educational level was also included as a covariate since food culture involves parental influence. Parental socioeconomic status, which refers to parent’s income, employment status and level of education, is a multifactorial concept of importance for growth, development, health outcomes and education of children [68, 69]. Socioeconomic status is a good predictor of dietary habits and quality of sleep [70–72], and may contribute to food-related practices [69]. Because parental education level likely influenced subjects’ dietary choices and sleep quality, parental education level was included as a covariate.

Physiological, environmental, and behavioral factors may offer some insight for the association between sleep and diet [63, 73–77], perhaps through changes in eating behaviors and patterns. The effects reported here likely reflect environmental and social factors (e.g., time constraints, work schedules, not preparing home meals). In Nogales specifically, most “non-Mexican” food outlets include fast food and quick-service restaurants, so infrequent consumption of Mexican food may reflect tendencies towards these options. Beyond their apparent lack of healthiness, frequent consumption of fast food is associated with lower levels of healthy behaviors and increased financial and time-related constrains on food choices. Therefore, the observed relationship between Mexican diet and sleep duration may reflect these socioeconomic pressures in addition to any biological mechanisms affected by the food itself. In other words, individuals who did not consistently eat Mexican food at home may have made more unhealthy food choices in general because of non-food-related demands. Thus, the relationship between sleep duration and diet may reflect a regional lack of variety in food options and/or other demands that are related to sleep duration. Unfortunately, specific eating habits were not assessed in this study, so future studies should evaluate environmental factors that may influence eating habits.

While food type and variety are known to affect sleep [1, 30, 78, 79], this study did not collect information on actual foods eaten or what foods were considered culturally-consistent by survey respondents. Mexican food culture is rich and is known to be protective against cardiometabolic disorders [50–54, 57–61], but there are limited data examining the relationship between Mexican food and sleep. Therefore, future work on sleep and diet should measure specific dietary data using a standardized food frequency questionnaire. Additional information on timing and type of food consumption on weekdays versus weekends would also be informative given the known associations of meal timing with sleep quality and duration [80–82] and since weekday stressors may be differ from weekend stressors.

Lastly, consuming culturally-consistent food was associated with a decreased likelihood of snoring. Snoring is associated with obstructive sleep apnea and the rates of sleep disordered breathing among individuals of Mexican descent is substantial. In a recent study by Li and colleagues [83], sleep disordered breathing was associated with 54% increased likelihood of hypertension and 33% increased likelihood of diabetes among the U.S. Hispanic/Latino population [24, 83]. This has important implications as sleep disorders are underrepresented, underreported, and undertreated among Hispanic/Latino populations. Thus, consuming culturally-consistent foods may provide a modifiable avenue for intervention for cardiometabolic disease prevention in this population.

### Strengths

This study has several strengths. The use of the ARSMA [43] quantified acculturation more comprehensively than the use of single proxy items (e.g. language immigration status), and the use of both Mexican and Anglo subscales provided a more complete understanding of participants’ acculturation. Another strength is that participants were recruited from the US-Mexico border in Arizona, a region which is rural and understudied compared to the urban population used in typical epidemiological research. Lastly, this study provided much needed information regarding border health disparities, particularly in sleep among individuals of Mexican descent.

### Limitations

There are also a few limitations worth mentioning. The cross-sectional study design precludes causal claims, so a longitudinal replication of this study could clarify the association found in this study. Also, despite being one of the largest studies to assess culturally-consistent diet and sleep, the present sample may be underpowered, resulting in unstable effects and wide confidence intervals. However, the non-significant findings has *p* values that were much greater than 0.05, suggesting small changes in the sample size would not impact the outcome much. Nonetheless, replication with a larger sample would strengthen these findings and could explore the influence of confounders, mediators/moderators and other unaddressed factors. Additionally, the use of self-report data means these findings are vulnerable to recall and reporting biases. Finally, sleep is related to a wide range of social, behavioral, and environmental factors, and the relatively small sample used here limited the number of covariates to only those deemed necessary. For this reason, health-related variables such as Body Mass Index, history of illnesses, medication use, and physical activity were not included because these factors are so closely related to both sleep and diet that they would over-control the regression models and preclude reasonable inferences. Future studies should consider assessing how these factors may contribute to sleep and dietary choices.

## Conclusion and future directions

The present study explored the relationship between culturally-consistent diet and its association with sleep duration, insomnia severity, daytime sleepiness, sleep quality, and snoring among adults of Mexican descent at the US-Mexico border. The primary findings were that consuming a culturally-consistent diet was associated with longer sleep duration and decreased likelihood of snoring, while there was no association with daytime sleepiness, insomnia, or sleep quality. Future studies should examine environmental, behavioral, and psychosocial factors that may influence these relationships, investigate potential protective factors stemming from cultural influences in the community, and identify possible modifiable strategies that may help prevent these risks.

## Data Availability

Not Applicable.

## Abbreviations

ARSMA-II: The Acculturation Rating Scale for Mexican-Americans II
ESS: Epworth Sleepiness Scale
ISI: Insomnia Severity Scale
PSQI: Pittsburgh Sleep Quality Index
AOS: Anglo-Orientation Scale
MOS: Mexican-Orientation Scale
